# Construction of physical maps for the sex-specific regions of papaya sex chromosomes

**DOI:** 10.1186/1471-2164-13-176

**Published:** 2012-05-08

**Authors:** Andrea R Gschwend, Wenli Zhang, Qingyi Yu, Paul H Moore, Jiming Jiang, Andrew H Paterson, Ray Ming

**Affiliations:** 1 Department of Plant Biology, University of Illinois at Urbana Champaign, Urbana, IL, 61801, USA; 2 Texas AgriLife Research Center, Weslaco, TX, 78596, USA; 3 Department of Horticulture, University of Wisconsin, Madison, WI, 53706, USA; 4 Plant Genome Mapping Laboratory, University of Georgia, Athens, GA, 30606, USA; 5 Hawaii Agriculture Research Center, Kunia, HI, 96759, USA; 6 Department of Agricultural Bio-resources, National Academy of Agricultural Science, RDA, Suwon, 441-701, Republic of Korea; 7 Department of Agronomy, University of Florida, Gainesville, FL, 32610, USA; 8 Departamento de Genética, Facultad de Ciencias, Universidad de Granada, Granada Campus de Fuentenueva s/n, 18071, Spain; 9 Shandong Agricultural University, Tai’an, Shandong, 271018, China; 10 Laboratory of Plant Developmental Genetics, Institute of Biophysics, Academy of Sciences of the Czech Republic, Kralovopolska 135, Brno, CZ-612 65, Czech Republic

**Keywords:** Bacterial artificial chromosome (BAC), *Carica papaya*, Sex chromosomes, Sex determination, Suppression of recombination

## Abstract

**Background:**

Papaya is a major fruit crop in tropical and subtropical regions worldwide. It is trioecious with three sex forms: male, female, and hermaphrodite. Sex determination is controlled by a pair of nascent sex chromosomes with two slightly different Y chromosomes, Y for male and Y^h^ for hermaphrodite. The sex chromosome genotypes are XY (male), XY^h^ (hermaphrodite), and XX (female). The papaya hermaphrodite-specific Y^h^ chromosome region (HSY) is pericentromeric and heterochromatic. Physical mapping of HSY and its X counterpart is essential for sequencing these regions and uncovering the early events of sex chromosome evolution and to identify the sex determination genes for crop improvement.

**Results:**

A reiterate chromosome walking strategy was applied to construct the two physical maps with three bacterial artificial chromosome (BAC) libraries. The HSY physical map consists of 68 overlapped BACs on the minimum tiling path, and covers all four HSY-specific Knobs. One gap remained in the region of Knob 1, the only knob structure shared between HSY and X, due to the lack of HSY-specific sequences. This gap was filled on the physical map of the HSY corresponding region in the X chromosome. The X physical map consists of 44 BACs on the minimum tiling path with one gap remaining in the middle, due to the nature of highly repetitive sequences. This gap was filled on the HSY physical map. The borders of the non-recombining HSY were defined genetically by fine mapping using 1460 F_2_ individuals. The genetically defined HSY spanned approximately 8.5 Mb, whereas its X counterpart extended about 5.4 Mb including a 900 Kb region containing the Knob 1 shared by the HSY and X. The 8.5 Mb HSY corresponds to 4.5 Mb of its X counterpart, showing 4 Mb (89%) DNA sequence expansion.

**Conclusion:**

The 89% increase of DNA sequence in HSY indicates rapid expansion of the Y^h^ chromosome after genetic recombination was suppressed 2–3 million years ago. The genetically defined borders coincide with the common BACs on the minimum tiling paths of HSY and X. The minimum tiling paths of HSY and its X counterpart are being used for sequencing these X and Y^h^-specific regions.

## Background

Most flowering plant species are hermaphrodites having both male and female sexual organs in the same flower. Only about 6% of flowering plant species are dioecious with male and female flowers on separate individual plants. Although the number of dioecious plants is small, they are widely distributed across different genera and families [1]. Some important dioecious crops include asparagus, spinach, poplar, kiwi fruit, strawberry, pepper, and papaya. It is proposed that the dioecious species have been evolved from ancestral hermaphrodite species along with the evolution of sex chromosomes [2]. Only a small number of plant sex chromosomes have been characterized at the cytological and molecular levels [3]. The dioecious plant species provide a unique opportunity for understanding the evolution of plant sex and sex chromosomes because of the co-existence of hermaphrodite, gynodioecious, androdioecious, dioecious, or even trioecious species and the possession in some families of sex chromosomes at various evolutionary stages [3,4].

Papaya is a trioecious species with three sex types: female, male, and hermaphrodite. Sex determination in papaya has long been a subject of genetic studies. Hofmeyr [5] and Storey [6] independently reported that sex in papaya was under the control of a single gene with three alleles: M, male; M^h^, hermaphrodite; and m, female. Any combination of the dominant alleles, MM, MM^h^, or M^h^M^h^, is lethal, resulting in heterogametic hermaphrodites (M^h^m) and males (Mm) and homogametic females (mm). More recently, high density genetic mapping of the papaya genome revealed severe recombination suppression at a sex determination locus [7]. Fine mapping and physical mapping of the sex determination locus validated suppression of recombination at the sex locus of 0.9– 2 Mb in length where no recombinant was detected [8]. Survey sequencing of selected BACs on the physical map revealed increased repetitive sequences and decreased gene density compared with genome wide averages. These findings led to the conclusion that sex determination in papaya is controlled by a pair of incipient sex chromosomes [8]. Integration of genetic and physical maps along with the genome sequence reveals that the suppression of recombination at the sex specific region. The recombination rates gradually resume to genome-wide average beyond the sex-specific region and then exceed the average by 7-fold, demonstrating the dynamics of recombination surrounding the HSY [9]. Similar incipient sex chromosomes were also found in the flowering plants Asparagus [10], poplar [11], and strawberry [12].

Male and hermaphrodite plants in papaya are controlled by two slightly different Y chromosomes, Y for males and Y^h^ for hermaphrodites, with sex chromosome genotypes XY and XY^h^, respectively. Female plants have a sex chromosome genotype XX [13]. There is a non-recombining male- or hermaphrodite-specific region of the Y or Y^h^ chromosome (MSY or HSY). The HSY was previously estimated to be about 4–5 Mb in size [8]. The sex chromosomes of papaya evolved about 2–3 million years ago estimated by time divergence analysis of four pairs of homologous X-Y^h^ genes [14]. The MSY and HSY shared 98.8% sequence identity including repetitive sequences, and these two Y chromosomes evolved from an ancestral Y chromosome about 73,000 years ago [14]. The X- and Y/Y^h^-specific sequences diverged extensively, sharing 84-86% sequence identity [14,15]. The two X only chromosomes are nearly identical, sharing 99.97% sequence identity [15]. Fluorescent *in situ* hybridization (FISH) reveals that the papaya HSY region spans about ~ 13% of the papaya Y^h^ chromosome with centromere embedded [16]. Five heterochromatic knob structures are detected in the HSY, and among them, Knobs 2, 3, 4, and 5 are HSY-specific and Knob 1 is shared between X and HSY. These five knobs contain heavily methylated DNA sequences. The centromere of Y^h^ chromosome appeared to be located on either side of Knob 4 [16].

Complete sequencing of the HSY and the corresponding X regions in papaya is highly desirable as it would unveil the genomic features and evolutionary events of young sex chromosomes in plants. The draft genome sequence of SunUp female plant provided the foundation for analyzing papaya genome composition, structure, and organization. It also facilitated the analysis of the papaya sex chromosomes [17]. The objectives of the present study are: (1) to construct separate physical maps of the HSY and its X counterpart with minimum tiling paths for sequencing the X- and Y^h^-specific regions of the papaya sex chromosomes; and (2) to define the borders of HSY genetically by fine mapping using SSR and SCAR markers around the border with large F_2_ populations.

## Results

### Physical mapping of HSY

Physical mapping started with screening the hermaphrodite BAC library using the sex co-segregating sequence characterized amplified region (SCAR) marker W11 by which four positive BACs were identified. The BAC end sequences (BES) of all four positive BACs were used for sorting out the configuration to select the two most extended ends as probes for the next round of screening of the BAC library. This stepwise chromosome walking process produced an HSY contig of about 900 Kb.

Additional sex co-segregating markers were used for screening the BAC library to establish multiple start points to extend the physical map, which greatly improved HSY chromosome walking that was hindered by highly repetitive sequences. Eighty five of the 225 sex co-segregating AFLP markers had sufficient distance from neighboring bands and fragment size (200 to 500 base pairs) to be suitable for Southern hybridization. These 85 markers were excised from AFLP gels to develop *Carica papaya* sex-liked markers (CPSM). Sixty three of the 85 fragments were re-amplified using the original AFLP primers. These 63 CPSMs were hybridized to the hermaphrodite BAC library, and 42 yielded positive hits. Among the 42 hybridized markers, 27 identified BACs that were either in the existing contig or formed new contigs. The remaining 15 probes hit numerous BACs indicating that they contained repetitive sequences. The library screening using CPSM resulted in five contigs with a combined size of 2.5 Mb [8].

Physical mapping was further assisted by sequencing selected seven BACs that were confirmed by FISH on the Y^h^[14,18]. These seven clones served as anchors, or start points, for chromosome walking to extend the physical map and to build the minimum tiling path. When the genome wide physical map based on fingerprints of the entire hermaphrodite BAC library became available [9], BACs on a minimum tiling path were selected and validated by multiple PCR amplification, and the BACs at the extreme ends of the existing contigs were used as seeds to search for contigs in the physical map. The genomewide physical map coupled with BAC end sequences (BES) accelerated construction of the HSY physical map. At this stage, higher stringency selection criteria were applied when adding new BAC clones: (1) primers from one of the BES of the candidate extension clone must amplify the anchor/seed BAC clone and vice versa; (2) at least two PCR product sequences show 100% match in the overlapping region between the candidate BAC and the established BAC in the physical map; (3) one or both BES of the candidate show male-specificity (not in all cases); (4) ultimately, candidate BAC clones have to be confirmed to be on the Y^h^ chromosome by FISH [16].

**Figure 1:**
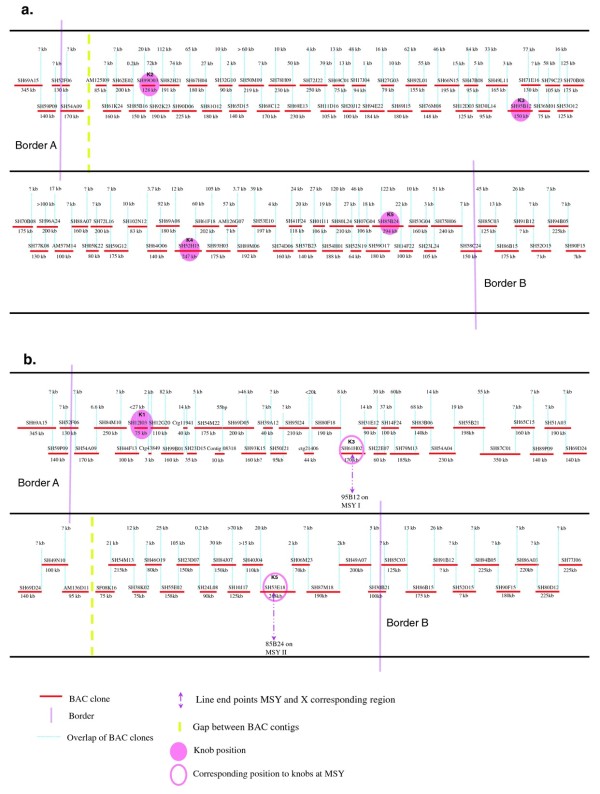
**The physical maps of the HSY and its corresponding X region with BAC clones on the minimum tilling path.** Red lines represent individual BAC clone with clone ID on the top and estimated insert size at thebottom. Blue dotted lines indicate the overlap of neighboring BAC clones. K1 ~ K5 indicate the position of heterochromatic knob-like structures [16]. Purple lines defined the borders of the non-recombination region genetically by fine mapping. SH: SunUp hermaphrodite BACs; SF: SunUp female BACs; AM: AU9 male BACs.

Four (Knobs 2 – 5) of the five heterochromatic knob structures that were Y^h^-specific [16] were positioned on the physical map (Figure1a). Unmapped Knob 1, the only Knob structure shared between the Y^h^ and X chromosomes, located in the gap between Knob 2 and Border A. Chromosome walking to close this gap kept landing to the X chromosome and thus could not fill the gap. The last two BACs at the end of the contig bearing Knob 2, SH62E02 and SH61K24, were confirmed by FISH to be on the X chromosome (Figure1a).

The physical map of HSY reached about 8.5 Mb from four contigs with the remaining three gaps. Multiple attempts to close the gaps by re-hybridization of probes from the extreme ends of contigs to the hermaphrodite BAC library were unsuccessful. Two new BAC libraries were constructed using different restriction enzymes, one from male AU9 and the other from female SunUp [19] for the screening. Two of the three gaps were filled by screening the male AU9 BAC library, one with AM126G07 and the other with AM57M14. A third male AU9 BAC, AM125I09, extended the contig for the remaining gap. This BAC was confirmed to be on the Y^h^ chromosome, even though the seed BAC 61K24 was on the X chromosome. The final version of the HSY physical map was now about 8.5 Mb, excluding shared border sequences with X chromosome (Figure1a).

A total of 332 PCR-amplified probes was used for library screening and primers used for designing probes were used again for further confirmation of positive clones and cross amplification for configuration during chromosome walking (Additional file 1: Table S1). Unfortunately, a majority of those positive clones from library screening had been eliminated due to less than maximal extension, redundancy, or more likely being false positive as a result of repetitive elements or the initial low stringency library screening. A minimum BAC tiling path containing 77 anchored BACs (Figure1a) was built on the basis of the several lines of evidence during chromosome walking as described in the methods section.

### Physical mapping of the HSY corresponding region in the X chromosome

Two X chromosome-specific BAC clones, SH53E18 (EF661026) and SH61H02 (EF661023), previously isolated through the presence of X-specific sequences, showed high sequence identity (>95%) to genic regions of two HSY BACs, SH 95B12 and SH85B24, respectively [14]. These two X BACs were used as seed BACs to start chromosome walking on the X counterpart on both directions simultaneously. New seed BACs were identified based on sex-co-segregating SSR markers from the draft genome of female SunUp [17,20,21]. For example, the source sequence of the sex-co-segregating SSR marker, P6K697Y0, aligned with the end sequences of SH30B21 with 99% identity, and the SH30B21 were used as new starting points for chromosome walking on both directions until overlapping BAC clones were identified or the gaps were filled. In addition to the basic chromosome walking steps applied for the HSY, we also applied *in silico* chromosome walking using sequence data from the draft genome of female papaya [17] for physical mapping of the X chromosome (in Material and Methods). BAC clones, SH12I03, SH12G20, SH99B01, and SH23D15 were all identified through *in silico* chromosome walking.

Two contigs were built with one gap remaining after exhausting the genomic resources, including the draft genome, physical map, and BES. Attempting to fill the gap ( Additional file 2: Supplemental note 1) was not successful. The remaining gap on the X physical map was closed on the HSY physical map. The gap of the HSY physical map between Border A and Knob 2 was filled on the X physical map. The BAC used to identify Knob 1 by FISH was mapped in the region near Border A of the X chromosome map.

The physical map of the X counterpart consists of 57 SH-BACs, including 13 border BACs shared between HSY and X. The minimum tiling path of the X-specific region contains 44 BACs, spanning about 5.4 Mb, and with a gap remaining between AM136D11 and SF08K16 (Figure1b).

### Physical mapping of borders A and B

Mapping the borders of the non-recombining HSY ensures the completeness of the HSY and its corresponding X physical maps. Two BACs, SH85C03 and SH86B15, were identified first through chromosome walking for the HSY physical map. Soon after, we identified the same extended BACs in the X chromosome walking process, suggesting that the location of this shared BAC clones might be in the border region. Concurrently, sex-linked SSR markers were used to identify more clones at the two border regions. Two sex-linked SSR markers (CPM1055C0 and P3K2608C0), showed genetic distances of 2 and 3 cM from the non-recombining HSY, respectively, one on each side the HSY. These two markers were used to retrieve the source sequences in the draft genome. The associated BAC clone SH94G23 was designated as border A and SH68N07 was designated as border B of the HSY region. BAC SH68N07 at border B was physically about 1 Mb away from SH86B15 and in the same fingerprint map as SH86B15 ( Additional File 3: Figure S2). Therefore, the minimal tiling path between these two BACs was established by the fingerprint map being confirmed by cross amplification among the overlapped BACs (Figure S2b). BAC SH94G23 at border A was almost imbedded in SH69A15, another large clone about 345 kb in size. SH69A15 was used as a seed BAC for chromosome walking in both directions until SH54A09 was identified, which overlapped with the X BAC clone, SH84M10 during chromosome walking. However, a gap remained unfilled between border A and the HSY physical map, specifically between SH54A09 and AM125I09, (Figure1a). Attempt to fill the gap had identified clones that were all on X chromosome without any evidence of being Y^h^-specific. This region contains Knob 1, the only Knob structure shared between X and HSY.

### Defining the borders of HSY via fine mapping

The method used to genetically define the HSY borders started with the sex-linked SSR marker CPM1055C0 on border A and P3K2608C0 on border B to search for recombinants between them among 1460 progeny of F_2_ population. Four recombinants were identified at the CPM1055C0 locus at border A only from the progeny of the cross AU9 x SunUp, because there were no polymorphic markers near CPM1055C0 in the other F2 populations. Eighty-seven recombinants were obtained for border B (Table1). The Kapoho x SunUp populations exhibited the highest rate of recombination rate at the P3K2608C0 locus with 7.7 % of the progeny, the AU9 x SunUp progeny had a recombination rate of 7.1 %, and the UH918 x SunUp Dwarf cross had the lowest at 2.6 %.

**Table 1:** Populations used for fine mapping

**No**	**Cross**	**Female**	**Hermaphrodite**	**Not determined**	**Total**
1	Keak Dum x 2H94 year1 Keak Dum x 2H94 year2	59 57	113 129	3 1	175 187
2	UH918 x SunUp Dwarf	52	144	3	199
3	AU9 x SunUp	19	79	0	98
4	Kapoho X SunUp	265	543	55	863
	Total	452	1008	62	1522

Seventy-five SSR markers (32 at border A and 43 at border B) were developed from the available BES and the draft genome sequences near the border regions to define the non-recombining region (Figure1, Table2, and [17]). Five of the 32 markers were polymorphic in the AU9 x SunUp mapping population, in order from the distal region of border A to the sex determining region: spctg177-12a, 84M10ctg-34a and -34b, 54 M22-BACs-636a, and 54 M22-BACs0-716b. The spctg177-12a marker identified two recombinants among 98 F_2_ progeny of the AU9 x SunUp family while the other four polymorphic markers co-segregated with sex. These results suggested that border A is located between spctg177-12a and 84M10ctg-34a/b, i.e. between BACs SH52F06 and SH84M10 (Figure1b; Table3).

**Table 2:** Sequences used for SSR marker development

**No**	**Sequence ID**	**Location**	**Sequence source**	**Length (bp)**	**Note**	**GB accession**
1	SH75H06	HSY	BAC	221878	Near border B	AC238771
2	SH58C24	HSY	BAC	148206	Near border B	AC239153
3	SH54M22	X	BAC	159764	Near Border A	AC239202
4	SH99B01	X	BAC	157433	Near Border A	AC238637
5	SH69D05	X	BAC	187502	Near Border A	AC239161

**Table 3:** The position of SSR markers for fine mapping and the number of recombinants in the populations

**SSR markers**	**Marker** location in BAC	**KhakDum x**2H94 year1	**KhakDum x**2H94 year2	**UH918 x** SunUp Dwarf	**AU9 x** SunUp	**Kapoho X** SunUp
CPM1055C0*	69A15	-	-	-	4	-
spctg177-12a	52 F06	-	-	-	2	-
84 M10ctg6-34a/b	84M10	-	-	-	0	-
54M22_BACs_662a	99B01	N/A	N/A	N/A	0	N/A
54M22_BACs_716b	99B01	N/A	N/A	N/A	0	N/A
…..						
P3K4642YC0*	53E18	0	0	0	0	0
58 C24-25b	58 C24	1	0	0	0	0
P3K8303YC0*	85 C03	1	0	0	0	2
BorderB-2001a	86B15	1	0	0	0	2
P3K2608C0*	68 N07	4	9	5	7	62

Two of 43 SSR markers: 58 C24-25b and BorderB-2001a were polymorphic at border B. BorderB-2001a identified three recombinants among the 87 previously identified ones. These three recombinants were then mapped using the 58 C24-25b marker which located inside BorderB-2001a. Only one recombinant was detected (Table3). No recombinations were observed at the position of a more inner sex-co-segregating marker, P3K4642, suggesting that border B was located between 58 C24–25b and P3K4642, within BAC SH58C24 (Figure1b; Table3). Therefore, the total HSY region was defined genetically to lie between BACs SH84M10 and SH85C03.

## Discussion

An important finding from the HSY and X physical maps is the rapid expansion of the HSY in the past 2–3 million years after genetic recombination became suppressed in the sex determining region. The HSY physical map contains about 8.5 Mb Y^h^-specific sequences with the distinctive cytological feature of having four Y^h^-specific knobs. In contrast, the HSY counterpart X is only 4.5 Mb in size, excluding the 900 kb region of Knob 1 which is shared with the HSY. The 89% expansion of physical size in HSY indicates rapid expansion of Y-specific sequences in the early stages of sex chromosome evolution. Given sufficient time, the X and Y may evolve into morphologically distinctive chromosomes, from each other and from autosomes. Thus the Y chromosome would become significantly larger than the X, as is the case with the young sex chromosomes of *Silene latifolia* that evolved about 10–20 million years ago [22]. Similarly, the medaka fish MSY exhibited 68.1% DNA sequence expansion [23] while the stickleback Y-specific BAC sequence showed 37% expansion compared with their X sequence [24]. Expansion of the Y chromosome in papaya has progressed until most gene content will be lost by the time when the Y starts a contraction phase of sex chromosome evolution [3].

The four Y^h^-specific heterochromatic knobs might account for a large portion of the HSY expansion, mostly by retrotransposon insertions, as evident by the 85% repetitive sequences in HSY versus 60% in its X counterpart [17]. The HSY is much larger than our original estimate of 4–5 Mb which was based on the 57% sex co-segregating sequence characterized amplified region (SCAR) markers embedded in the then 2.5 Mb physical map of HSY [8]. The AFLP derived SCAR markers were developed from markers that were polymorphic between the female and hermaphrodite parents. These polymorphic and sex co-segregating markers might be enriched in Y^h^ specific sequences, such as the four Knobs, causing the underestimate of the physical size of HSY.

We could not identify a region in the papaya X chromosome that corresponds to the Knob 2 in HSY. Moreover, the distance between Knob 1 and the corresponding region of Knob 3 on the X chromosome was much shorter than the distance between Knobs 2 to 3 on HSY. Knob2, represented by SH99O03 on HSY, was highly HSY-specific, indicating accumulation of novel sequences after recombination was suppressed by retrotransposon insertions, translocations, and perhaps other chromosomal rearrangements.

The mapping of border BACs that are not distinguishable between X and Y^h^ allowed us to develop additional markers from the draft genome and from sequencing of selected BACs to genetically define the borders of HSY. Four F_2_ populations with a total of 1522 individuals were analyzed for SSR markers near the borders to identify recombinants. Only seven of the 77 SSR markers developed were polymorphic for the four pairs of parent papaya cultivars (Table1). At border A, all five polymorphic markers were found in only one (AU9 x SunUp) of the four F2 populations. Our finding of more polymorphism at border A in the AU9 x SunUp family than in the others is supported by the report that SunUp and AU9 are more distantly related than are the other cultivars [25]. A total of 87 recombinants were found at border B from initial screening using the SSR marker P3K2608. A significant reduction of recombination was observed in all populations near the border B region between P3K2608 (in BAC SH68N07, outside of border B, not on the physical maps of HSY and X) and BorderB-2001a (in BAC SH86B15), a distance of 1.6 Mb. Our finding of very little recombination in all populations reinforces the numerous reports that sex determination regions are characterized by suppression of recombination. The HSY region was genetically defined to lie between the two SSR markers, spctg177-12a in Border A BAC SH52F06 and 58 C24–25b in Border B BAC SH58C24.

Despite numerous attempts, even when utilizing an additional male AU9 BAC library, the gap between border A and HSY remained unfilled. Chromosome walking on HSY kept jumping to land on the X chromosome. This 900 Kb region is where Knob 1 is located, the only knob structure shared between the X and Y^h^ chromosomes [16]. It is likely that the jump from HSY to X while walking is due to higher DNA sequence homology between the X and Y^h^ at Knob 1 than in other parts of the chromosome due to the paucity of Y^h^-specific sequences near Knob 1. For these reasons, the region of Knob 1 could be beyond the HSY, even though we were not able to detect any recombination in this region. Suppression of recombination in this region could be the results of heterochromatic sequence in Knob 1. This gap on the HSY was filled on the physical map of the X counterpart.

For a genomic region like the HSY that is rich in repetitive sequences, it is unusual that the 8.5 Mb physical map of HSY could be assembled into only one contig. This success might have been facilitated by the large average insert size of 174 Kb of the second batch of the hermaphrodite SunUp BAC library [26]. This possibility is apparent by noting that the BACs mapped on the HSY and X physical map frequently contain large inserts (often above 200 Kb) (Figure1).

Although there is no gap in the border regions of the physical map of the X counterpart there is one within the map. We made substantial efforts to close this gap using two additional BAC libraries, one from male AU9 and one from female SunUp. We also used the draft genome of female SunUp [17], but the gap remained. When comparing the physical maps and knob locations of HSY and X, it appears that the gap within the X physical map may correspond to Knob 4 of HSY (Figure1). Based on cytological FISH results, HSY BAC SH52H15 of Knob 4 mapped to the most terminal position compared to the other four knobs at metaphase I. Authors of that report suggested that the centromere of the Y^h^ chromosome is located between Knobs 4 and 5 [16]. The gap we were unable to fill on the physical map of the X counterpart could be the centromere of the X chromosome. Interestingly, this gap was filled on the physical map of HSY, so it is possible that we have fine mapped its centromere. If so, this is a surprise as most centromeres are highly repetitive and heterochromatic, very difficult to map or sequence, as has been repeatedly reported by genome sequencing projects of multiple organisms [27,28]. To date, the centromere of rice chromosome 8 (*Cen8*) is the only reported sequenced centromere in plants [29]. Rice *Cen8* contains active genes and is at an early stage of centromere evolution, evolving from a neocentromere to a mature centromere. If the centromere of the Y^h^ chromosome is indeed between Knobs 4 and 5, we may have been able to fine map it because it is atypical, perhaps a recently evolved centromere.

Complete sequencing of the papaya HSY and its X counterpart is the first step towards identification of the sex determination gene(s), which could lead to engineering a true breeding hermaphrodite variety without the Y^h^ chromosome. In addition, the sequences of papaya sex chromosomes would be the first one in plants, and the third Y chromosome to be sequenced including the Y chromosomes of humans and the chimpanzee [30,31]. Physical mapping of a region without recombination is a challenging task, and our task was further confounded by a potentially embedded centromere and highly repetitive, heterochromatic sequences. The trioecious nature of papaya and genomic resources already developed for it expedited our sequencing efforts. It was possible to construct an HSY and corresponding X map only through having the three BAC libraries representing the three sex types of papaya, a collection of BES from the hermaphrodite BAC library, a genome wide physical map, and an annotated draft genome [9,17,19,21,26]. FISH validation proved crucial to ensure that we were walking on the correct molecule.

## Conclusions

We applied a reiterate chromosome walking strategy and constructed the physical maps for the heterchromatic HSY and its X counterpart. The non-recombination region between HSY and X was defined genetically by fine mapping to ensure the complete coverage of these two physical maps in papaya. The HSY expanded rapidly comparing to it X counterpart in 2 – 3 million years. The previously identified five heterochromatic knob structures were all covered, four in the map of HSY and one in the map of X counterpart. These two physical maps will serve as templates for complete sequencing of the sex determination region in papaya. The sequencing of HSY and its X corresponding region is being completed using the minimum tiling path of these two physical maps.

## Methods

### BAC libraries

The three BAC libraries used for physical mapping were constructed from leaves of: (1) a hermaphrodite plant of the gynodioecious papaya variety SunUp (SH) [26], (2) a female plant of SunUp (FS), and (3) a male plant of the dioecious variety AU9 (AM) [19]. The hermaphrodite SunUp BAC library, as the primary one used in this study, included 39,168 BAC clones providing 13.7X papaya genome equivalents. This library was from *Hin*d III digestion with an average insert size of 132 kb from two batches of partial digestion; the first batch consisted of 18,432 BAC clones with an average insert size of 87 kb, The second batch consisted of 20,736 BAC clones with an average insert size of 174 kb [26]. The second batch of the hermaphrodite SunUp BAC library was particularly useful for physical mapping by providing many large-insert clones that were used to build the minimum tiling path for sequencing. Two additional BAC libraries were constructed using different restriction enzymes to fill the possible gaps of the primary BAC library. *Bst*Y I was used for the SunUp female BAC library which consisted of 36,864 clones with an average insert size of 104 kb, providing 10.3x genome equivalents. *Eco*R I was used for the AU9 male BAC library which consisted of 55,296 clones with an average insert size of 101 kb, providing 15.0x genome equivalents [19].

### BAC end cloning and BAC end sequencing

A total of 67,179 BES of the hermaphrodite SunUp BAC library was obtained through high throughput BAC DNA isolation and sequencing of both ends of the BAC DNA with ABI 3700 x1 DNA Analyzers [21]. We applied both BAC end cloning and BAC DNA direct sequencing for the BAC clones with failed BES or without BES for all libraries including the hermaphrodite SunUp, female SunUp, and male AU9. In the BAC end cloning procedure, 30 ng of BAC DNA were isolated through standard miniprep procedure and digested with *Not* I and *Dra* I and then ligated to *Not I* and *EcoRV* sites of pPCR-script AMP SK(+) vector (Stratagene). The BAC ends were directly amplified by using KS (5′-TCGAGGTCGACGGTATCG-3′) and LF1 (5′-ACCTGCAGGCATGCA-AGC-3′) primers for left end, and KS and RR4 (5′-GGTGACACTATAGAATACTCAAGC-3′) primers for right end. The amplified BAC end with a size between 200–1000 bp was purified and sequenced by ABI 3730XL. For the BAC DNA direct sequencing approach, the BAC DNA was isolated using Pure Yield Plasmid Midiprep System (Promega, Madison, WI, USA) following the manufacturer’s instructions. The DNA concentration was adjusted to 200 ng/ul and then sequenced by the ABI 3730XL using T7 for the left BES and M13rev primers for the right.

### Primer design

Primer3 (v. 0.4.0) [32] was used for designing primers used for chromosome walking. The default criteria for the primers designed from available BES was applied except that the product size range was adjusted to reach the maximum allowed by the size of the BES. About 60 bp at the start and end of the sequences were avoided for primer design due to possible poor sequence quality. For the primers designed from fully-sequenced BAC clones, about 5 kb of sequence from each end were submitted to Primer3 and three pairs of primers with product size between 800-1000 bp were designed at 1, 2, and 3 kb away from the ends. All primers were synthesized by Invitrogen Corp (Carlsbad, CA 92008 USA) and Bioneer Inc (Alameda, CA 94501).

### BAC library screening through hybridization

The PCR products (1–5 ug) used as probes were purified with Wizard SV gel and PCR clean-up (Promega, Madison, WI, USA) and eluted to 16 μl with double distilled water. The probes labeled with digoxigenin-dUTP were used for hybridization, and the hybridization signal was detected by chemiluminescence method using DIG High Prime DNA Labeling and Detection Starter Kit II (Roche Applied Science, Indianapolis, IN 46250 USA). The procedure was mainly based on the manufacturer’s instructions with modification. Briefly, the 20 μl labeling reaction including the 16 μl denatured probe and 4 μl DIG-High Primer was incubated at 37°C for 20–24 hrs. The filter (23 x 23 cm) sets containing the complete BAC library gridded in duplicate were pre-hybridized overnight in Q-trays with 100 ml of DIG Easy Hyb buffer and then hybridized overnight in fresh buffer including denatured labeled probe and 50 ng labeled λ DNA for blocking background signals. After hybridization, the filters were subjected to high stringency washes and immunological detection according to the instruction and exposed to X-ray film for 3–12 hrs. Positive BAC clones were identified by BAC-DMS 2.1, a SAS coded program [33].

### BAC library screening through PCR

Clones of the hermaphrodite SunUp BAC library were pooled following the Matrix and SuperPool pooling strategy by Amplicon Express (Pullman, WA 99163 USA). The pool screening was done in a two-round PCR procedure by first using super pools (12 plates) as templates and second using matrix pools as template following the User’s Manual version 3.2 (Amplicon Express, Pullman, WA 99163 USA). Identification of positive clones was accomplished by comparing the positive hits to the pool keys provided in the manual.

### PCR confirmation of positive clones and configuration

The positive clones identified from the library screening were inoculated into 200 μl Luria broth (LB) medium with 12.5 μg/μl of chloramphenicol and grown overnight at 37°C on a rotary shaker. The glycerol stocks were made by mixing equal volumes of overnight culture and 30% glycerol. The stocks were used as templates for PCR to verify the positive clones from library screening and to configure the extent of overlap with the existing physical map. The 10 μl PCR reaction included 0.8 μl glycerol stock of the clone, 1X PCR buffer, 0.15 mM of each dNTP, 1.8 mM MgCl_2_, 0.15 μM of reverse and forward primers, and 0.8 units of *Taq* polymerase. The PCR program was 94°C for 5 min, then 40 cycles of 30 s of denaturing at 94°C, 40 s of annealing at 55°C, and 30–60 s (depending on the expected size) of extension at 72°C, and then with a final extension at 72°C for 10 min. PCR products were separated on 1.0 % agarose gel and visualized on a gel doc system (BioRad, Hercules, CA 94547 USA).

### Y-specificity test

For Y-specificity test of PCR-confirmed positive clones, PCR was run on all templates including glycerol stocks of the positive clones and four DNA samples from female SunUp, hermaphrodite SunUp, female AU9, and male AU9 as templates. If the size of the PCR products from the male or hermaphrodite were distinctively different from that of the females of SunUp and AU9, the marker was documented as Y-specific.

### Fluorescence *in situ* hybridization

The HSY locations of the extended positive BAC clones were further validated by Fluorescence *in situ* hybridization (FISH). Slide preparation was described previously [16]. The FISH procedure was conducted according to Jiang et al. (1995)[34].

### BAC insert size determination

The confirmed positive BAC clones were inoculated into 5 mL of LB medium with 12.5 μg/μl chloramphenicol and incubated overnight at 37°C on a rotary shaker. Plasmid DNAs were isolated from the culture using a standard miniprep procedure. After precipitation, the DNA pellet was disolved in 30 μl of TE buffer. 10 μl of the plasmid DNA was subjected to *Not I* enzyme digestion at 37°C for 3 hr and loaded to the Clamped homogenous electric fields (CHEF) gel with the MidRange Marker II (New England Biolab, USA) as a size standard.

### Chromosome walking to identify the extended clones

Chromosome walking was conducted in both directions until an overlapped clone was identified or gaps between contigs were filled. The basic steps employed during chromosome walking to identify each extended BAC clones from the anchored clones are outlined in Additional file 4: Figure S1.

For chromosome walking on X counterpart, we also applied *in silico* chromosome walking using sequence data from the draft genome of female papaya [17]. We blasted the BESs of anchored BACs against the sequence contigs of the female papaya draft genome. The top hits with sequence identity above 98% were then blasted against the BESs of all the clones in the hermaphrodite BAC library [21]. The candidate overlapping BAC clones were identified and then subjected to PCR confirmation and cross amplification to sort out the configuration of the BAC contigs. The remaining steps were the same as for HSY chromosome walking to validate overlapping sequences ( Additional file 4: Figure S1). FISH was also used to examine the localization of each potential anchor BAC clone on the X chromosome.

### Genomic resources

Relevant genomic resources include a draft genome of female SunUp [17], the BES of a hermaphrodite SunUp BAC library [21], a physical map of the papaya genome [9], and a collection of 16,362 EST unigenes [17].

### Fine mapping of the sex determining region

The F_2_ mapping populations of 1522 progenies were derived from four different crosses (Table1). These populations were grown at the Kunia substation on Oahu, Hawaii. Young leaves of each F_2_ progeny were used for DNA isolation [35]. Among the 1460 individuals, 452 were phenotyped as female and 1008 as hermaphrodite by observing their flowers types, and 62 were excluded from the analysis due to unknown (not scored) sex type.

SSRs longer than 18 bp in length and the corresponding primers for each SSR were identified using SSR finder software (http://www.maizemap.org/bioinformatics.htm) and used for fine mapping. The source sequences for SSR primer design from the draft genome of female SunUp included HSY BACs (SH75H06 and SH58C24), X BACs (SH54M22, SH99B01, SH69D05), BACs near borders (SH12I03, SH84M10, and SH86B15), and 4 supercontigs (66, 177, 123, 39). All SSR primers ( Additional file 5: Table S2) were synthesized by Bioneer Inc (Alameda, CA 94501). The primer sets were screened for polymorphism using DNA samples of the parents and two DNA pools of female and hermaphrodite F_2_ progenies. The PCR conditions were described previously [20]. The markers exhibiting sex-linked polymorphism were used for fine mapping.

## Misc

Jong-Kuk Na and Jianping Wang contributed equally to this work.

## Competing interests

The authors declare that they have no competing interests.

## Authors’ contributions

JKN, JW, JEM, AG, QY, CC, and ZK carried out physical mapping. JW and JKN participated in experimental design of physical mapping. JW, JKN, and RM wrote the manuscript; WZ performed FISH analysis for BAC localization on sex chromosome; RNP and FAF participated in sequence analysis and reviewed the manuscript. RM, QY, PHM, JJ and AHP conceived of the study, coordinated, and organized all research activities. All authors read and approved the final manuscript.

## Supplementary Material

Additional file 1 **Table S1.**Summary of probes for BAC library screening and the initial screening results. Click here for file

Additional file 2 Supplemental note 1. Click here for file

Additional file 3 **Figure S1.**The working map indicating the overlaps through cross amplification. Click here for file

Additional file 4 **Figure S2.**The work flow chart illustrating the basic steps of chromosome walking on HSY physical mapping. Click here for file

Additional file 5 **Table S2.**List of SSR primers used for fine mapping. Click here for file
