# Supplementary material for: Construction of physical maps for the sex-specific regions of papaya sex chromosomes
Source: BMC Genomics. 2012 May 8;13:176. doi: 10.1186/1471-2164-13-176 (PMC3430574; doi:10.1186/1471-2164-13-176)
Supplement: Additional file 2 — Supplemental note 1. [file 1471-2164-13-176-S2.docx]

Supplemental note 1:

Two additional BAC libraries, male AU9 (containing one X chromosome) and female SunUp (containing two X chromosomes), were screened using probes from the ends of the gap to close the only remaining gap of the X physical map [21]. A probe designed from the end sequence of BAC SH49N10 hybridized to 15 BACs from hermaphrodite, 96 from female, and 41 from male BAC libraries. PCR amplification using primers designed from the end of SH49N10 confirmed that SH49L10, SF13E17 and SF33E10, and AM136D11 were positive. The sequences of the amplicons were 100% match to the end sequences of SH49N10. We sequenced the ends of these positive clones and used primers from the BESs to cross-amplify SH49N10 and themselves to configure the orientation of these clones. However, only the primers designed from AM136D11 end sequences amplified SH49N10. The other three potential positive clones were classified as false positives, perhaps caused by repetitive sequences at the end of SH49N10. We used FISH to further validate the location of AM136D11 within the X region corresponding to the HSY. However, AM136D11 hybridized to multiple spots and the major signals were not in either HSY or the corresponding X region. Assuming that the end sequences of AM136D11 may be highly similar to the true sequence at this location, the BES of AM136D11 were blasted against the female draft genome sequence which identified contig 38610 of 2235bp with 100% match. A probe designed from this contig was hybridized to all three BAC libraries, identifying in 90 BACs from hermaphrodite, 25 from female, and 83 from male BAC libraries. PCR amplification confirmed 10 positive BAC clones, and the sequences of the amplicons matched the end sequence of AM136D11 at 98%, which could be expected since AM136D11 might not be the direct target sequence. FISH mapping of these 10 BACs showed repetitive hybridization signals without any strong signal at the target region (Figure 1b). The male AU9 and female SunUp BAC libraries were used to extend the other end of the gap. A BAC from the female SunUp BAC library, SF08K16, overlapped with SH54M13, but FISH mapping also showed repetitive hybridization signals.
