# Supplementary material for: Construction of physical maps for the sex-specific regions of papaya sex chromosomes
Source: BMC Genomics. 2012 May 8;13:176. doi: 10.1186/1471-2164-13-176 (PMC3430574; doi:10.1186/1471-2164-13-176)
Supplement: Additional file 3 — Figure S1.The working map indicating the overlaps through cross amplification. [file 1471-2164-13-176-S3.ppt]

## Slide 1
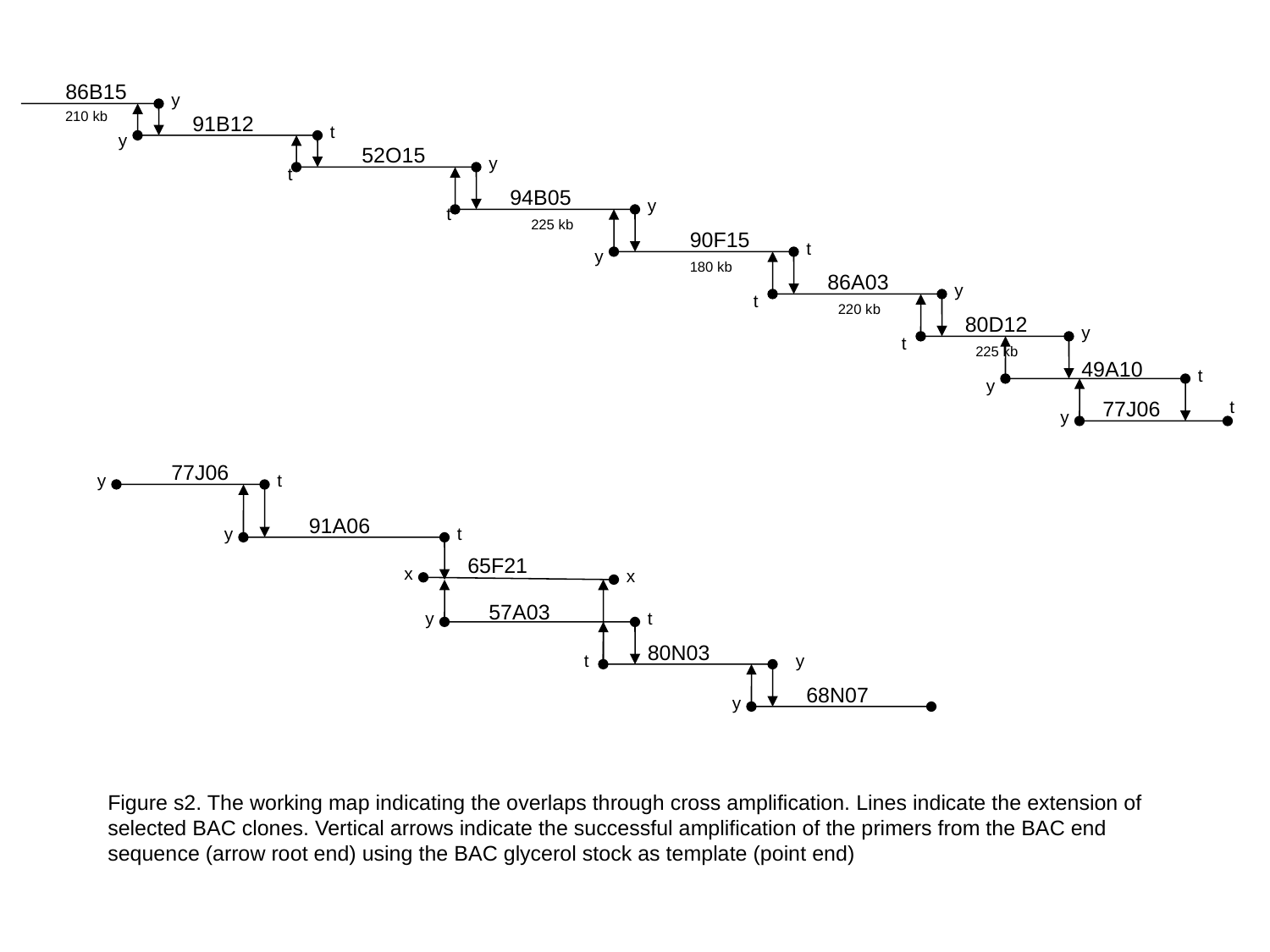

86B15
y
210 kb
91B12
t
y
52O15
y
t
94B05
y
t
225 kb
90F15
t
y
180 kb
86A03
y
t
220 kb
80D12
y
t
225 kb
49A10
t
y
77J06
t
y
77J06
y
t
91A06
y
t
65F21
x
x
57A03
y
t
80N03
t
y
68N07
y
Figure s2. The working map indicating the overlaps through cross amplification. Lines indicate the extension of selected BAC clones. Vertical arrows indicate the successful amplification of the primers from the BAC end sequence (arrow root end) using the BAC glycerol stock as template (point end)
