# Supplementary material for: Construction of physical maps for the sex-specific regions of papaya sex chromosomes
Source: BMC Genomics. 2012 May 8;13:176. doi: 10.1186/1471-2164-13-176 (PMC3430574; doi:10.1186/1471-2164-13-176)
Supplement: Additional file 4 — Figure S2.The work flow chart illustrating the basic steps of chromosome walking on HSY physical mapping. [file 1471-2164-13-176-S4.ppt]

## Slide 1
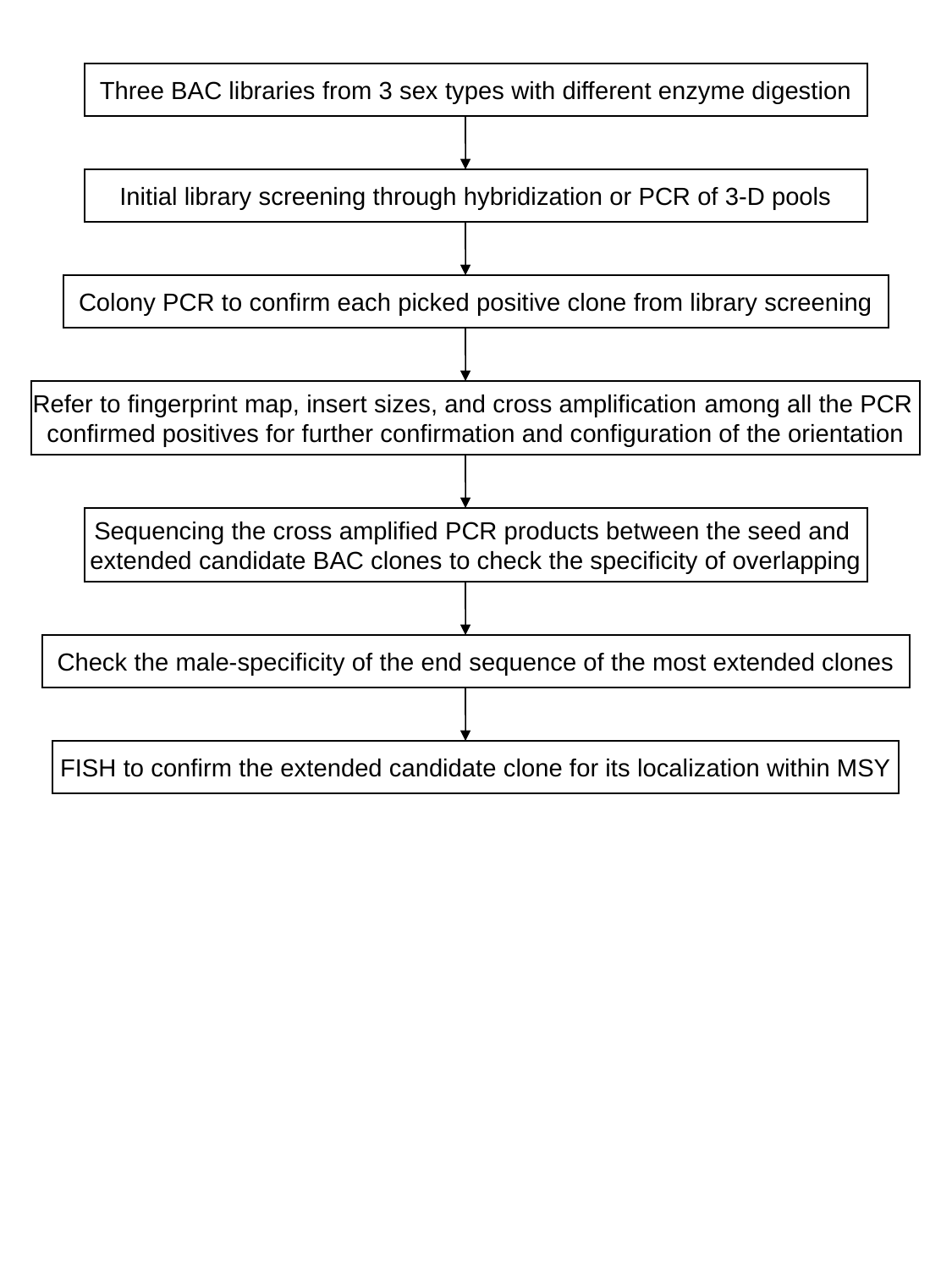

Three BAC libraries from 3 sex types with different enzyme digestion
Initial library screening through hybridization or PCR of 3-D pools
Colony PCR to confirm each picked positive clone from library screening
Refer to fingerprint map, insert sizes, and cross amplification among all the PCR
confirmed positives for further confirmation and configuration of the orientation
Sequencing the cross amplified PCR products between the seed and
extended candidate BAC clones to check the specificity of overlapping
Check the male-specificity of the end sequence of the most extended clones
FISH to confirm the extended candidate clone for its localization within MSY
